# Dequalinium chloride promotes the killing efficacy of aminoglycoside antibiotics against *Pseudomonas aeruginosa* by enhancing membrane potential

**DOI:** 10.1128/spectrum.04183-25

**Published:** 2026-09-09

**Authors:** Xiaoya Wei, Zhonghui Yang, Yujie Zhang, Xin Xia, Zhihui Cheng, Yongxin Jin, Shouguang Jin, Weihui Wu

**Affiliations:** 1 State Key Laboratory of Medicinal Chemical Biology, Key Laboratory of Molecular Microbiology and Technology of the Ministry of Education, Department of Microbiology, College of Life Sciences, Nankai University 12538, Tianjin, China; The University of Texas at Tyler, Tyler, Texas, USA

**Keywords:** dequalinium chloride, *Pseudomonas aeruginosa*, aminoglycoside antibiotics

## Abstract

Dequalinium chloride is a dicationic amphiphilic quaternary ammonium compound that is used as an anti-infective drug. Here, we found that it potentiates bactericidal efficacies of aminoglycoside antibiotics against *Pseudomonas aeruginosa*. Dequalinium chloride increases respiratory activity and bacterial membrane potential, thereby promoting intracellular uptake of aminoglycosides. Furthermore, the dequalinium chloride–amikacin combination demonstrated a synergistic bactericidal effect in a murine acute pneumonia model, indicating its therapeutic potential.

**IMPORTANCE:**

Antimicrobial resistance (AMR) poses a severe global health threat. Novel antibiotics and therapeutic strategies are urgently needed. This study revealed that dequalinium chloride (DC) synergizes with aminoglycosides against planktonic and biofilm-associated *P. aeruginosa*, which is an important pathogen. Mechanistic study reveals that DC enhances bacterial respiration and membrane potential to promote aminoglycosides uptake. The DC–amikacin combination is effective in a murine pneumonia model. These findings provide a promising therapeutic strategy for combating *P. aeruginosa* infections.

## OBSERVATION

Antimicrobial resistance (AMR) has become a critical global health threat (1). It is critical to develop new antimicrobials and treatment strategies. Antibiotic combination therapy has been widely used in clinics. The advantages include synergistic effects, delaying resistance development, and expanding antimicrobial coverage (2, 3).

Dequalinium chloride (DC) is a double-headed broad-spectrum cationic antimicrobial agent that exerts potent bactericidal activity through disrupting bacterial cell membrane integrity and interfering with cellular metabolism. Clinically, DC is widely used in oral lozenges for pharyngeal infections and vaginal tablets for bacterial vaginosis (4, 5). As an amphiphilic molecule, dequalinium has been employed as a vehicle for the inhaled delivery of curcumin to treat lung injury (6).

*Pseudomonas aeruginosa* is a gram-negative opportunistic pathogen that frequently causes hospital-acquired infections in immunocompromised individuals (7, 8). In this study, we investigated the effect of DC on *P. aeruginosa* in a physiologically relevant medium (95% Roswell Park Memorial Institute medium [RPMI] + 5% Mueller–Hinton broth [MHB]), which offers a closer resemblance of the host environment compared to nutrient-rich laboratory media, such as the Mueller–Hinton broth (MHB) (9). We further evaluated the combinatorial effects between DC and various types of antibiotics.

**Fig 1:**
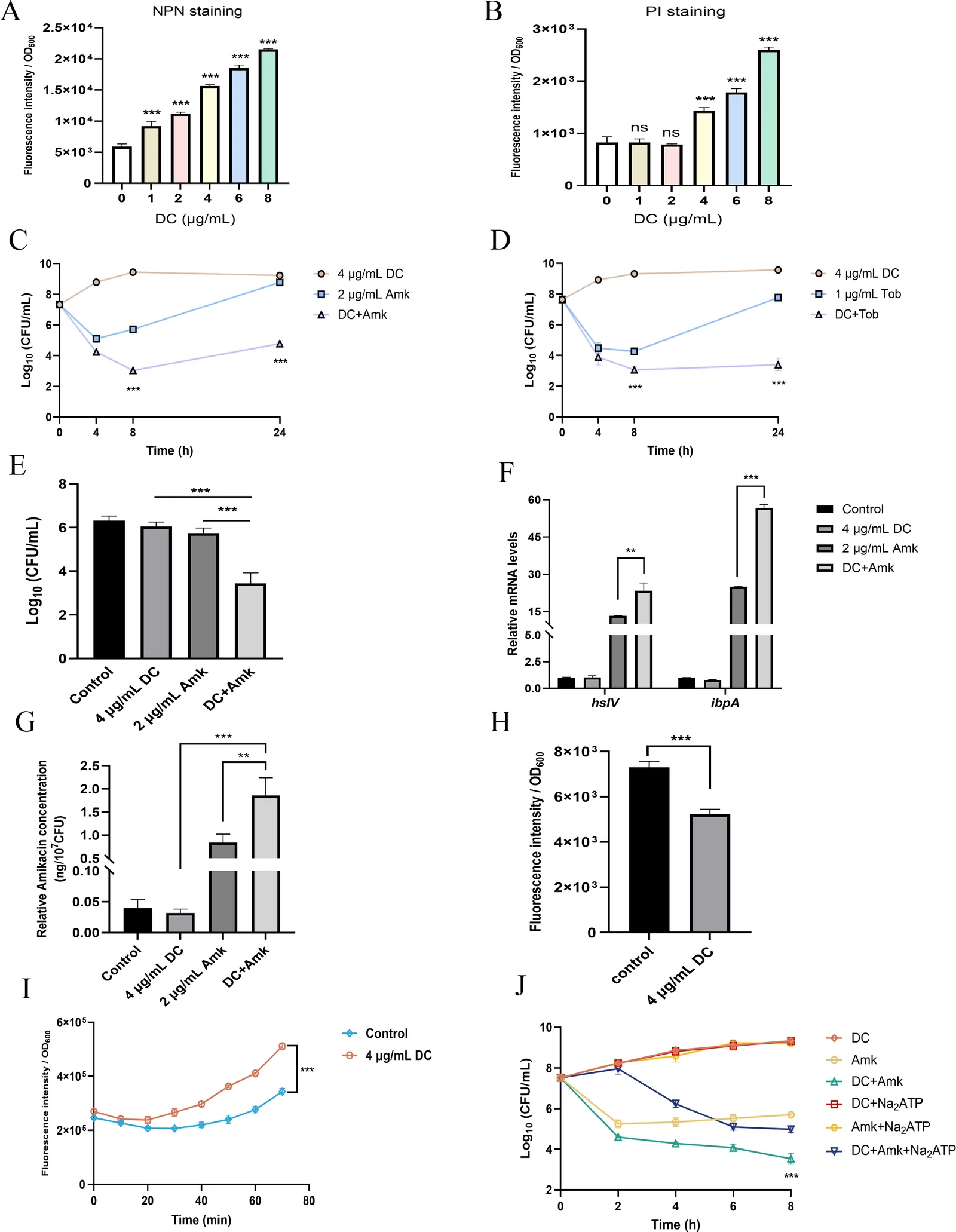
Synergistic bactericidal effect of DC with aminoglycoside antibiotics against PA14. NPN (**A**) and PI (**B**) staining following DC treatment. ns, not significant; ***, *P* < 0.001 compared to bacteria without DC treatment. (**C and D**) Bactericidal effects of 4 μg/mL DC in combination with 2 μg/mL amikacin (**C**) or 1 μg/mL tobramycin (**D**) at indicated time points. ***, *P* < 0.001 compared to the single-antibiotic treatment group at the corresponding time points. (**E**) Killing effects of 4 μg/mL DC and 2 μg/mL amikacin against biofilm-associated bacteria. ***, *P* < 0.001. (**F and G**) Wild-type PA14 was treated with DC (4 μg/mL) and amikacin (2 μg/mL) alone or in combination for 1 h at 37°C. (**F**) The mRNA levels of *hslV* and *ibpA* genes were determined by RT-qPCR. (**G**) Intracellular amikacin amounts were determined by ELISA. **, *P* < 0.01; ***, *P* < 0.001 compared to bacteria treated with amikacin alone. (**H and I**) Wild-type PA14 was treated with or without 4 μg/mL DC for 1 h. (**H**) Bacterial membrane potential was assessed by staining with DiSC_3_(5). (**I**) Cellular respiratory activity was evaluated via Alamar Blue staining. ***, *P* < 0.001 compared to the bacteria without DC treatment. (**J**) Wild-type PA14 was treated with 4 μg/mL DC and 2 μg/mL amikacin alone or in combination in the presence or absence of 10 mM Na_2_ATP at 37°C. At indicated time points, viable bacterial counts were quantified via plating. ***, *P* < 0.001 compared to the bacteria without Na_2_ATP treatment. Data in **A, B, E, **and **G **were analyzed by a one-way ANOVA with Dunnett’s (**A** and **B**) or Bonferroni’s (**E** through **G**) *post-hoc* tests. Data in **C, D, F,** and **J **were analyzed by a two-way ANOVA with Bonferroni’s *post-hoc* test. Data in **H** and** I **were analyzed by a two-tailed Student’s *t*-test. Data represent the mean ± standard deviation of the results from three samples. DC, dequalinium chloride; Amk, amikacin; Tob, tobramycin.

In CA-MHB, the MIC of DC for a reference strain PA14 is higher than 128 μg/mL, whereas that is 2 μg/mL in the physiologically relevant medium (Table S2). Since DC is positively charged, we speculated that it might interact with the negatively charged LPS and affect the outer membrane integrity. N-phenyl-1-naphthylamine (NPN) and propidium iodide (PI) staining revealed that at the concentrations of 1 and 2 μg/mL, DC impaired outer membrane integrity without damaging the inner membrane, whereas at 4 μg/mL or higher concentrations, DC caused damages to both inner and outer membranes (Fig. 1A and B).

Since impairment of the membrane might promote the influx of antibiotics, we examined whether DC synergizes with commonly used antibiotics against *P. aeruginosa*. The concentration of DC was used at 4 μg/mL, which is the lowest concentration that impairs outer and inner membrane integrity (Fig. 1A and B). Amikacin, ciprofloxacin, and meropenem were used at concentrations lower than their corresponding clinical breakpoint (10). Azithromycin was used at 4 μg/mL, which is lower than the MIC for PA14 (64 μg/mL) in the CAMHB (11). Tigecycline was used at the lowest concentration (16 μg/mL) required to inhibit 90% of *P. aeruginosa* clinical isolates (12). Among the tested antibiotics, DC synergized with amikacin, resulting in approximately 9.3 × 10^3^ fold more killing than amikacin alone 24 h after the treatment (Fig. 1C; Fig. S1 and Table S2). We then tested the bactericidal effect of DC in combination with another aminoglycoside antibiotic, tobramycin, which also displayed synergistic effect (Fig. 1D and Table S2). A checkerboard assay demonstrated a Fractional Inhibitory Concentration Index (FICI) of 0.625 between amikacin and DC against PA14 (Fig. S2).

The *P. aeruginosa* biofilm is highly resistant to antibiotics and host immune clearance compared to its planktonic form, leading to persistent or recurrent infections (13–15). Compared to amikacin alone, the DC–amikacin combination resulted in 153-fold more killing of biofilm-associated bacteria (Fig. 1E). These results demonstrate a synergistic effect of DC and amikacin against planktonic and biofilm-associated PA14.

Given that DC at the concentration of 4 μg/mL impaired membrane integrity without killing the bacteria (Fig. 1A through D), we suspected that DC might promote the killing effect of amikacin by promoting its influx. The intracellular aminoglycoside amounts have been shown to correlate with the expression of heat shock protein genes, including *ibpA* and *hslV* (16). RT-qPCR results demonstrated that DC enhanced the amikacin-induced upregulation of those genes (Fig. 1F). In addition, enzyme-linked immunosorbent assay (ELISA) results verified that DC increased intracellular accumulation of amikacin (Fig. 1G).

It has been established that the membrane potential produced by the electron transport chain (ETC) is essential for the uptake of aminoglycoside antibiotics (17). DiSC_3_(5) staining demonstrated that DC increased membrane potential (Fig. 1H) (18). In addition, the Alamar Blue assay demonstrated that DC enhanced bacterial respiration activity (Fig. 1I).

To verify whether the DC-enhanced respiration promoted the uptake of amikacin and subsequent bactericidal effects, we treated the wild-type PA14 with a TCA cycle inhibitor Na_2_ATP, which had been shown to decrease membrane potential (19). The presence of Na_2_ATP increased bacterial survival following treatment with the DC–amikacin combination (Fig. 1J). Collectively, these results demonstrate that DC promotes the uptake of amikacin by enhancing membrane potential.

**Fig 2:**
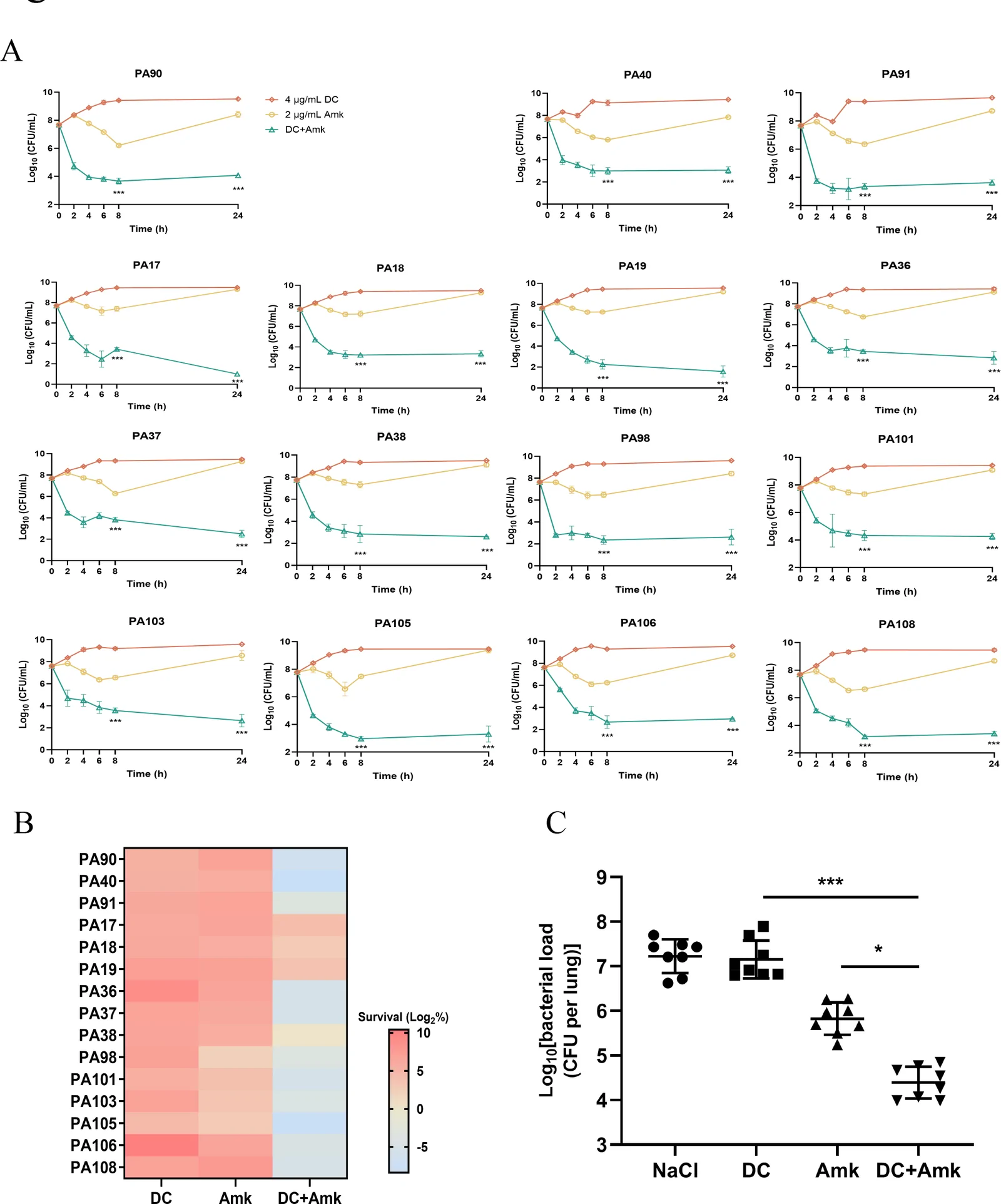
Bactericidal activities of the DC-amikacin combination against *P. aeruginosa in vitro* and *in vivo*. (**A**) Clinical isolates were treated with DC (4 μg/mL) and amikacin (2 μg/mL) alone or in combination at 37°C. At indicated time points, viable bacterial counts were quantified via plating. **, *P* < 0.01; ***, *P* < 0.001 compared to the single-antibiotic treatment groups at the corresponding time points by a two-way ANOVA with Bonferroni’s *post-hoc* test. Data represent the mean ± standard deviation of the results from three samples. (**B**) Biofilm of the 15 clinical strains was treated with DC (4 μg/mL) and amikacin (2 μg/mL) alone or in combination for 24 h. Survival data were normalized to the untreated control group (100% survival baseline). Log_2_-transformed biofilm survival percentages were shown as a heatmap. (**C**) Each female mouse was infected intranasally with 4 × 10^6^ CFU of wild-type PA14. After 3 h, infected mice were administered intranasally with DC and amikacin alone or in combination. Bacterial loads were determined 13 h after the treatment. *, *P* < 0.05; ***, *P* < 0.001 compared to the single-antibiotic treated groups by a Kruskal–Wallis test with Dunn’s *post-hoc* test. DC, dequalinium chloride; Amk, amikacin.

Next, we evaluated the killing efficacies of DC in combination with amikacin against 15 clinical isolates (Table S3). The MICs of DC and amikacin were all greater than or equal to 4 and 2 μg/mL, respectively (Table S3). After 24 h, the combined treatment increased the killing efficacies by more than 2 × 10^4^-fold compared to amikacin alone (Fig. 2A). We then examined the bactericidal effects against the biofilm of the clinical isolates. Compared to amikacin alone, the combined treatment increased the killing efficacies by 206–1.5 × 10^4^ fold against 10 clinical strains (Fig. 2B; Fig. S3). As for strains #98 and #38, the combination resulted in 66- and 46-fold more killing compared to amikacin alone, respectively (Fig. 2B; Fig. S3). Among the remaining three strains (#17, #18, #19), no monotherapy displayed bactericidal activity, whereas the combination treatment resulted in 5–10-fold killing (Fig. 2B; Fig. S3). Overall, these results demonstrate that the DC–amikacin combination was effective against planktonic and biofilm associated *P. aeruginosa* strains.

To investigate whether pulmonary surfactant affects the bactericidal effect, we performed a time-kill assay in a physiologically relevant medium containing 5 g/L mucin, which was based on the “CF-like” medium (20). After 24 h, the combined treatment increased the killing efficacies by more than 4 × 10^5^ -fold compared to amikacin alone (Fig. S4), demonstrating a synergistic effect.

To assess the *in vivo* efficacy of the combined therapy, we utilized a murine acute pneumonia model (21, 22). Immunocompetent female BALB/c mice were infected intranasally with wild-type PA14. At 3 h post-infection (hpi), the mice were administered intranasally with either vehicle control (normal saline), DC, amikacin alone, or in combination. The dose of amikacin was used at 35 mg/kg, which was in accordance with a prior study that employed the same pneumonia model (23). A previous study demonstrated that the maximum tolerated dose of DC in BALB/c mice was 15 mg/kg after a single intraperitoneal administration (24). In this study, DC was administered intranasally at 5 mg/kg to minimize potential side effects. Treatment with DC alone did not reduce the bacterial load, and amikacin alone reduced the average bacterial load by approximately 1.40 log_10_. However, the combination therapy reduced the average bacterial load by 2.83 log_10_. (Fig. 2C), demonstrating a synergistic bactericidal effect.

Overall, by using a physiologically relevant medium, we demonstrated that the cationic DC disrupts membrane integrity in a dose-dependent manner. Compared to MHB, growth in the physiologically relevant medium resulted in a transcriptome more similar to *P. aeruginosa* strains directly isolated from patients (9). Thus, MIC or bacterial killing assay in physiologically relevant medium may more closely represent the efficacies *in vivo*. Here, we found that DC enhances aminoglycoside uptake by increasing bacterial respiration and membrane potential. Previous studies demonstrated that bacteria increase respiratory activity and membrane potential in response to stresses, such as bactericidal antibiotics (23, 25), presumably aiming to repair stress-caused damages. The combination of DC with amikacin synergistically eradicates planktonic and biofilm-associated bacteria in both reference and clinical strains. The checkerboard assay demonstrated an additive effect between DC and amikacin (Fig. S2). The checkerboard assay evaluates growth inhibitory effect, in which bacteria were inoculated at a low concentration (5 × 10^5^ CFU/mL). However, the time-kill assay evaluates bactericidal effect, in which 5 × 10^7^ CFU/mL bacteria were treated with drugs. The DC–amikacin combination also demonstrated a synergistically bactericidal effect against *P. aeruginosa* in a murine pneumonia model. Optimizing the delivery method, such as inhaled formulation, may improve pulmonary bioavailability while reducing systemic side effects. Additionally, the mechanism of DC-enhanced bacterial respiration remains to be elucidated. Further *in vitro* and *in vivo* passage experiments are warranted to investigate the effect of DC on the development of bacterial resistance to amikacin. Among quaternary ammonium compounds, benzalkonium chloride was found to synergize with gentamicin against *P. aeruginosa*, whereas cetrimonium bromide antagonizes with gentamicin (26). These results indicate different effects of quaternary ammonium compounds on bacteria, which differentially affect the bactericidal effect of aminoglycoside antibiotics. Altogether, our results suggest that nebulized DC–amikacin combination might be beneficial in treating *P. aeruginosa*-caused pulmonary infections in humans.

## Data Availability

Data will be made publicly available upon publication and upon request for peer review.

## SUPPLEMENTAL MATERIAL

Supplemental materials spectrum.04183-25-s0001.docx Fig. S1 to S4, Tables S1 to S3, and additional experimental details.

ASM does not own the copyrights to Supplemental Material that may be linked to, or accessed through, an article. The authors have granted ASM a non-exclusive, world-wide license to publish the Supplemental Material files. Please contact the corresponding author directly for reuse.
