# Supplementary material for: Dequalinium chloride promotes the killing efficacy of aminoglycoside antibiotics against Pseudomonas aeruginosa by enhancing membrane potential
Source: Microbiol Spectr. 2026 Sep 9;14(10):e04183-25. doi: 10.1128/spectrum.04183-25 (PMC13688868; doi:10.1128/spectrum.04183-25)
Supplement: Supplementary file 1 [file spectrum.04183-25-s0001.docx]

**Supplemental materials**

**Bacterial strains and culture conditions**

The bacterial strains used in this study are listed in supplementary Table S1. *P. aeruginosa* strains were grown in Luria Broth (LB) at 37 ºC with shaking at 200 rpm.

**Minimal inhibitory concentration (MIC) test**

The antimicrobial susceptibility assay for all compounds were performed following the Clinical and Laboratory Standards Institute (CLSI) guidelines by broth microdilution in CA-MHB (cation adjusted-Mueller Hinton broth) or physiologically relevant medium (95% RPMI + 5% MHB) (1, 2). Dequalinium chloride, meropenem and tigecycline were purchased from Shyuanye (China). Amikacin, ciprofloxacin and azithromycin were purchased from Meilunbio (China), BBI (China) and Macklin (China), respectively.

**Outer membrane integrity assay**

The bacterial outer membrane integrity was assessed using the NPN staining assay (3). Briefly, bacterial cells in mid-logarithmic phase (OD_600_=0.5) were harvested and resupended in fresh physiologically relevant medium. The bacteria were incubated at 37 ºC for 1 hour with or without dequalinium chloride at the concentrations of 1, 2, 4, 6 or 8 μg/mL. The bacteria were then washed three times and resuspended in 5 mM GHEPES buffer (Caisson Labs) supplemented with 5 mM glucose. The fluorescent probe NPN was added at a final concentration of 10 μM and incubated at 25 ºC for 30 min, followed by measurement of fluorescence intensity with a Varioskan Flash fluorometer (Agilent) set at 350 nm for excitation and 420 nm for emission. The intracellular NPN levels were normalized to corresponding bacterial OD_600_.

**Inner membrane integrity assay**

The bacterial inner membrane integrity was assessed using the propidium iodide (PI) staining assay (4). Briefly, bacterial cells in mid-logarithmic phase (OD_600_=0.5) were harvested and resuspended in fresh physiologically relevant medium. The bacteria were incubated at 37 ºC for 1 h with or without dequalinium chloride at the concentrations of 1, 2, 4, 6 or 8 μg/mL. Cells were then washed three times and resuspended in the phosphate buffered saline (PBS). Subsequently, the fluorescent probe PI was added to the cell suspension to a final concentration of 10 μM and incubated at 25 ºC for 30 min. Then the PI fluorescence intensity was determined using a Varioskan Flash fluorometer (Agilent) set at 535 nm for excitation and 615 nm for emission. The intracellular PI levels were normalized to corresponding bacterial OD_600_.

**Time-dependent killing test**

The overnight culture of *P. aeruginosa* was diluted 1:100 in fresh LB and grown continuously at 37 ºC with shaking until OD_600_=1.0. The bacteria were then diluted 10-fold in the physiologically relevant medium with or without 5 g/L mucin, and treated with dequalinium chloride or the indicated antibiotic, either alone or in combination. At the indicated time points, samples were serially diluted in NaCl. 10 μL dilutions were spotted on LB agar, and bacteria colonies were counted after incubation overnight at 37 ºC. The experiment was performed in triplicate.

**Biofilm killing assay**

*P. aeruginosa* cells were grown in LB broth at 37 ºC with agitation at 200 rpm until OD_600_ reached 1.0. The bacterial suspension was adjusted to OD_600_=0.025 in fresh LB. 150 μL of bacterial suspension was added into each well of a 96 well plate and incubated at 37 ºC. After 24 hours, the bacterial solution was removed and physiologically relevant medium containing 4 μg/mL dequalinium chloride, 2 μg/mL amikacin, alone or in combination was added into each well. After 24 hours, the biofilm was washed three times and resuspended with 150 μL normal saline. The biofilm was dispersed by ultrasound and the bacterial counts were quantified by plating. The experiment was performed in triplicate.
**Checkerboard assay**

The fractional inhibitory concentration index (FICI) was determined by the checkerboard microdilution method in 96‑well plates according to CLSI guidelines (1). The checkerboard was performed in the physiologically relevant medium. In brief, two-fold serial dilutions of amikacin and DC were combined in a two-dimensional array, with concentration ranges covering 1/32 to 2× MIC of amikacin and 1/64 to 2× MIC of DC. Bacterial inoculum was adjusted to 5×10^5^ CFU/mL. 100 μL bacterial suspension was innoculated into each well of a 96-well plate, follwed by incubation at 37 ºC for 20 h. The percentage of inhibition for each well was calculated using the following formula:

Inhibition (%) = [(OD_600_ of positive control-OD_600_ of sterility control)-(OD_600_ of test well-OD_600_ of sterility control)] / (OD_600_ of positive control-OD_600_ of sterility control) × 100%

FICI was calculated as: FICI = (MIC of A in combination / MIC of A alone) + (MIC of B in combination / MIC of B alone). Effects were interpreted as: synergy (FICI ≤ 0.5), additive (0.5 < FICI ≤ 1.0), indifference (1.0 < FICI ≤ 4.0), or antagonism (FICI > 4.0) [PMID: 12805255]. All experiments were performed in triplicate.

**RNA isolation and quantitative real-time PCR (RT-qPCR)**

Bacteria were grown to an OD_600_ of 0.5 in LB and resuspended in fresh physiologically relevant medium, followed by incubation with 4 μg/mL dequalinium chloride, 2 μg/mL amikacin, either alone or in combination, for 1 hour at 37 ºC. The bacteria were collected by centrifugation and washed once with normal saline. Total bacterial RNA was purified using a Bacterial Total RNA Kit (Zoman Biotec, Beijing, China). 1 μg RNA was reverse-transcribed at 55 ºC using random primers and the PrimeScript Reverse Transcriptase (TransGen Biotech, Beijing, China). The resulting cDNA was amplified with gene-specific primers (see Supplementary Table S1) and SYBR Premix (TransGen) in a Fluorescent Quantitative PCR Detection System (Bioer, Hangzhou, China). The *rpsL* gene, encoding the 30S ribosomal protein, was used as the internal reference gene for data normalization.

**Amikacin uptake assay**

The wild type PA14 were cultured to an OD_600_ of 0.5 in LB medium, resuspended in the physiologically relevant medium, and treated for 1 hour with 4 μg/mL dequalinium chloride, 2 μg/mL amikacin, alone or in combination. After the treatment, a small portion of the bacteria was used to determine live cell numbers. The remaining cells were washed and resuspended with PBS, followed by lysis by sonication. Then the supernatant was collected after centrifugation at 12,000 rpm for 10 min at 4 ºC.

Intracellular amikacin was quantified using an indirect competitive Enzyme-linked immunosorbent assay (ELISA) according to the manufacturer's instruction (MlBio, Shanghai, China). Briefly, 50 µL of serial diluted standards (provided by the kit) or bacterial supernatant was added to each well of a 96-well plate, followed by addition of 50 µL of primary antibody. The plate was sealed with a cover membrane and incubated at 37 ºC for 30 min. Then each well was washed three times with 300 μL wash buffer. Subsequently, 100 µL horseradish peroxidase (HRP) conjugated secondary antibody solution was added to each well and incubated at 37 ºC for 30 min in dark. The plate was washed four times with 300 μL washing buffer, followed by sequential addition of 50 µL chromogen A and B. After incubation at 37 ºC for 15 min in dark, the reaction was stopped with 50 µL stop solution. The absorbance was measured at 450 nm using a Varioskan Flash fluorometer (Agilent) within 5 min. Intracellular amikacin levels were normalized by live bacterial numbers.

**Membrane potential assay**

Membrane potential was determined using 3, 3-Dipropylthiadicarbocyanine iodide [DiSC_3_(5)] as previously described (5). Bacteria were grown to OD_600_=0.5 at 37 ºC. The bacteria were resuspended in fresh physiologically relevant medium and incubated with or without 4 μg/mL dequalinium chloride for 1 hour. Then the bacteria were washed three times with 5 mM GHEPES buffer (Caisson Labs) containing 5 mM glucose and resuspend in the same buffer. DiSC_3_(5) was added at a final concentration of 2 μM. Fluorescence intensity was measured at 622 nm for excitation and 670 nm for emission with a Varioskan Flash fluorometer (Agilent). The change in membrane potential was normalized to bacterial OD_600_.

**Bacterial respiratory rate assay**

Bacterial respiratory rate was determined with alamarBlue as previously described (6). Bacteria were grown in LB at 37 ºC to an OD_600_ of 0.5. The bacteria were harvested, and resuspended in the physiologically relevant medium. 180 μL of the bacterial culture were mixed with 20 μL alamarBlue dye with or withour 4 μg/mL dequalinium chloride in each well of a black 96-well plate. The fluorescence was monitored using an excitation wavelength of 560 nm and an emission wavelength of 590 nm with a fluorometer (Varioskan Flash; Agilent) every 10 min for 70 min.

**Ethic statement**

The animal infection experiments were performed in compliance with the national guidelines for the use of laboratory animals. The protocol received approval from the Animal Care and Use Committee of the College of Life Sciences, Nankai University (permission number: NK-04-2392012).

**Murine lung infection model**

The acute pneumonia model was established in mice following previously reported procedures (7, 8). The wild type PA14 was cultured in LB to an OD_600_ of 1.0. Bacterial cells were washed twice with 0.9% NaCl and resuspended to a final concentration of 2 × 10⁸ CFU/mL. Four groups of immunocompetent female BALB/c mice (6–8 weeks old; Vital River), each consisting of eight animals, were anesthetized with isoflurane inhalation. Each mouse was infected 20 μL of the bacterial suspension via intranasal inoculation. At 3 hours post-infection (hpi), the mice were re-anesthetized and administered intranasally with 20 μL of one of the followings: 0.9% NaCl (vehicle control), dequalinium chloride (5 mg/kg), amikacin (35 mg/kg), or a combination of both antibiotics at the same doses used in the monotherapy groups. After 13 hours, all mice were euthanized by CO_2_ asphyxiation. The lungs were harvested and homogenized in sterile 1% proteose peptone (Solarbio, Beijing, China). Bacterial loads in the lungs were quantified by serial dilution and plating on agar plates.

**Statistical analyses**

All experiments were performed at least three times, and data are presented as mean ± standard deviation (SD), unless otherwise specified. All statistical analyses were performed using GraphPad Prism 10 (GraphPad Software Inc, USA). For single comparisons between two groups, a two-tailed Student’s t-test was used. For multiple comparisons at a single time point or concentration, data were analyzed using one-way analysis of variance (ANOVA), followed by Dunnett’s test or Bonferroni’s test. For data obtained at several time points or concentrations, two-way ANOVA was performed, followed by Bonferroni’s test. In the animal experiments, the non - parametric Kruskal - Wallis test with Dunn's post hoc test was used. Bacterial CFU counts were log_10-_transformed prior to analysis. Asterisks on graphs indicate significant differences, and the corresponding p-values were described in the figure legends.

**Supplemental figures**

**Figure. S1**

Fig S1. Bactericidal effects of dequalinium chloride in combination with indicated antibiotics at the indicated time points against PA14. ns, not significant; *, P < 0.05; ***, P < 0.001 compared to the single-antibiotic treatment groups at the corresponding time points by a two-way anova with Bonferroni’s post hoc test. DC, dequalinium chloride; Cip, ciprofloxacin; Mem, meropenem; Azm, azithromycin; Tgc, tigecycline.

**Figure. S2**

Figure S2. Checkerboard FICI heatmap for the combination of DC and amikacin against PA14. The X- and Y axes represent the serial two-fold dilutions of amikacin and DC, respectively. The color gradient indicates the relative percentage of inhibition at each combination.

**Figure. S3**

Fig S3. The bactericidal effects of dequalinium chloride (4 μg/mL) in combination with amikacin (2 μg/mL) against *P. aeruginosa* biofilm. **, P < 0.01; ***, P < 0.001 by a one-way anova with Bonferroni’s post hoc test. Data represent the mean ± standard deviation of the results from three samples. DC, dequalinium chloride; Amk, amikacin.

**Figure. S4**

Figure S4. The bactericidal effects of dequalinium chloride in combination with amikacin at the indicated time points against PA14 in the presence of mucin. ***, P < 0.001 compared to the single-antibiotic treatment groups at the corresponding time points by a two-way anova with Bonferroni’s post hoc test. DC, dequalinium chloride; Amk, amikacin.

**Supplemental tables**

**Table S1.** Bacterial strains and primers used in this study

| Strain | Description | Source |
| --- | --- | --- |
| P. *aeruginosa* |  |  |
| PA14 | Wild type strain of *P. aeruginosa* | (9) |
| CI-PA-17 | Clinical isolate | This study |
| CI-PA-18 | Clinical isolate | This study |
| CI-PA-19 | Clinical isolate | This study |
| CI-PA-36 | Clinical isolate | This study |
| CI-PA-37 | Clinical isolate | This study |
| CI-PA-38 | Clinical isolate | This study |
| CI-PA-40 | Clinical isolate | This study |
| CI-PA-90 | Clinical isolate | This study |
| CI-PA-91 | Clinical isolate | This study |
| CI-PA-101 | Clinical isolate | This study |
| CI-PA-103 | Clinical isolate | This study |
| CI-PA-105 | Clinical isolate | This study |
| CI-PA-106 | Clinical isolate | This study |
| CI-PA-108 | Clinical isolate | This study |
| Primers | Sequence 5’-3’ | Function |
| *rpsL*-RT-S | CAAGCGCATGGTCGACAAGAG | qRT-PCR |
| *rpsL-*RT-AS | ACCTTACGCAGTGCCGAGTTC | qRT-PCR |
| *ibpA*-RT-S | CCGCTGTTCCGTCATTCC | qRT-PCR |
| *ibpA*-RT-AS | GTCGTCACCGTGCTTTTCG | qRT-PCR |
| *hslV*-RT-S | GACCACCATCGTATCTGTCCG | qRT-PCR |
| *hslV*-RT-AS | TTCTTGGCGTTGCCTTTCA | qRT-PCR |

**Table S2.** MICs (mg/L) of indicated antibiotics for the PA14 strain

| Antibiotics**^#^** | Physiologically relevant medium | CAMHB |
| --- | --- | --- |
| DC^a^ | 2 | >128 |
| Amk^a^ | 1 | 2 |
| Tob^a^ | 0.5 | 0.5 |
| Cip^a^ | 0.125 | 0.25 |
| Mem^a^ | 0.25 | 0.5 |
| Azm^a^ | 0.5 | 128 |
| Tgc^a^ | 8 | 32 |

^#^ | All experiments were performed in triplicate.

^a^ | DC, dequalinium chloride; Amk, amikacin; Tob, tobmycin; Cip, ciprofloxacin; Mem, meropenem; Azm, azithromycin; Tgc, tigecycline.

**Table S3.** Clinical isolates used in this study

| Strain^#^ | Isolation sites | MIC (mg/L) | | | | Carbapenemase |
| --- | --- | --- | --- | --- | --- | --- |
|  |  | Physiologically relevant medium | | CAMHB | |  |
|  |  | DC^a^ | Amk^a^ | DC^a^ | Amk^a^ |  |
| CI-PA-17 | Burn wound secretions | 4 | 4 | >16 | 2 | KPC |
| CI-PA-18 | Burn wound secretions | 4 | 4 | >16 | 2 | KPC |
| CI-PA-19 | Burn wound secretions | 4 | 4 | >16 | 2 | KPC |
| CI-PA-36 | Burn wound secretions | 8 | 2 | >16 | 2 | KPC |
| CI-PA-37 | Burn wound secretions | 8 | 4 | >16 | 2 | KPC |
| CI-PA-38 | Burn wound secretions | 8 | 4 | >16 | 2 | KPC |
| CI-PA-40 | Burn wound secretions | 8 | 4 | >16 | 4 | KPC |
| CI-PA-90 | Sputum | 8 | 4 | >16 | 2 | KPC |
| CI-PA-91 | Venous catheter tip | 16 | 4 | >16 | 4 | KPC |
| CI-PA-98 | Burn wound secretions | 16 | 4 | >16 | 2 | KPC |
| CI-PA-101 | Burn wound secretions | 16 | 4 | >16 | 2 | KPC |
| CI-PA-103 | Burn wound secretions | 16 | 4 | >16 | 2 | KPC |
| CI-PA-105 | Burn wound secretions | 16 | 4 | >16 | 2 | KPC |
| CI-PA-106 | Burn wound secretions | 16 | 4 | >16 | 2 | KPC |
| CI-PA-108 | Burn wound secretions | 16 | 4 | >16 | 2 | KPC |

^#^ | All experiments were performed in triplicate. The clinical isolates were obtained from non-CF patients in Tianjin Forth Central Hospital.

^a^ | DC, dequalinium chloride; Amk, amikacin.

**Supplemental References**

1.	James S. Lewis II P, FIDSA. 2025. Performance standards for antimicrobial susceptibility testing, M100, 35th ed. Clinical and Laboratory Standards Institute.

2.	Belanger CR, Lee AH, Pletzer D, Dhillon BK, Falsafi R, Hancock REW. 2020. Identification of novel targets of azithromycin activity against *Pseudomonas aeruginosa* grown in physiologically relevant media. Proc Natl Acad Sci U S A 117:33519-33529.

3.	Helander IM, Mattila-Sandholm T. 2000. Fluorometric assessment of gram-negative bacterial permeabilization. J Appl Microbiol 88:213-219.

4.	Yarlagadda V, Akkapeddi P, Manjunath GB, Haldar J. 2014. Membrane active vancomycin analogues: a strategy to combat bacterial resistance. J Med Chem 57:4558-4568.

5.	Xia Y, Cebrian R, Xu C, Jong A, Wu W, Kuipers OP. 2021. Elucidating the mechanism by which synthetic helper peptides sensitize *Pseudomonas aeruginosa* to multiple antibiotics. PLoS Pathog 17:e1009909.

6.	Jackson AA, Gross MJ, Daniels EF, Hampton TH, Hammond JH, Vallet-Gely I, Dove SL, Stanton BA, Hogan DA. 2013. Anr and its activation by PlcH activity in *Pseudomonas aeruginosa* host colonization and virulence. J Bacteriol 195:3093-3104.

7.	Pan X, Wu W. 2020. Murine Acute Pneumonia Model of *Pseudomonas aeruginosa* Lung Infection. Bio Protoc 10:e3805.

8.	Ren H, Liu Y, Zhou J, Long Y, Liu C, Xia B, Shi J, Fan Z, Liang Y, Chen S, Xu J, Wang P, Zhang Y, Zhu G, Liu H, Jin Y, Bai F, Cheng Z, Jin S, Wu W. 2019. Combination of Azithromycin and Gentamicin for Efficient Treatment of *Pseudomonas aeruginosa* Infections. J Infect Dis 220:1667-1678.

9.	Liberati NT, Urbach JM, Miyata S, Lee DG, Drenkard E, Wu G, Villanueva J, Wei T, Ausubel FM. 2006. An ordered, nonredundant library of *Pseudomonas aeruginosa* strain PA14 transposon insertion mutants. Proc Natl Acad Sci U S A 103:2833-2838.
